# Estimating the causal effect of sleep duration on mental health in young adults: A meta-learner approach from physiological and nutritional perspectives

**DOI:** 10.1371/journal.pone.0345018

**Published:** 2026-04-02

**Authors:** Hak Gyun Oh, Jeong In Park, Ah Kyung Kim, Wah Wah Maw, Yerin Choi, Won Sang Lee

**Affiliations:** 1 Department of Data Science, Gangneung Wonju National University, Gangneung, Republic of Korea; 2 Department of Data Science, College of Computing, Data Science, and Society, University of California, Berkeley, California, United States of America; 3 Department of Data Science, Gangneung Wonju National University, Gangneung, Republic of Korea; King Abdulaziz University Faculty of Medicine, SAUDI ARABIA

## Abstract

This study analyzes the causal effect of sleep duration on mental health among young adults using a meta-learner-based causal inference framework. Specifically, we applied a T-Learner model with Random Forest and XGBoost as the base learner to data from 1,405 individuals aged 19–34, drawn from the 2022–2023 Korea National Health and Nutrition Examination Survey. The result indicates that adequate sleep increases the probability of maintaining normal mental health. Subgroup analysis comparing individuals with adequate sleep and normal mental health to those with insufficient sleep and poor mental health also reveals a statistically significant causal effect of sleep on mental health improvement. In addition, AST (SGOT) levels and blood creatinine concentration are identified as key confounding factors. Findings suggest that sufficient sleep could enhance mental health among young adults, and policy implications for youth mental health are derived from the perspective of sleep duration. By providing empirically identified causal evidence based on nationally representative data, this study contributes to the growing literature on sleep and mental health and highlights sleep duration as a modifiable target for evidence-based mental health interventions in young adults.

## 1. Introduction

Recently, the mental health among young adults has increasingly attracted the interest of academia and practitioners [1]. While the psychological problems of young adults, such as anxiety and depression, have accordingly emerged as a social issue, the lack of sleep time could be one of the important factors closely associated with these psychological conditions [2,3]. Young adults have increasingly experienced cognitive and emotional overload stemming from extensive engagement with SNS, mobile device usage, and social stress [4]. Those factors could worsen sleep deprivation, which seems to exert a pronounced adverse effect on mental health [1]. Given the diverse aspects of these factors, this study examines the causal effect of sleep deprivation on mental health, accounting for potential confounders. Focusing on South Korean young adults—who are notably exposed to high levels of stress, intensive media consumption, and widespread mobile technology—this study aims to empirically examine the mental health consequences of sleep deprivation in terms of these excessive stressors.

2024 Sleep Status Report on Koreans, published by the Korean Society of Sleep Research, reveals that the average sleep duration of Koreans is 6 hours and 58 minutes—18% less than the OECD average [5]. Around 60% of respondents reported experiencing sleep problems, while only 7% indicated they regularly have restful sleep, compared to the global average of 13%. The average sleep duration among young Koreans is just 6 hours and 7 minutes, indicating a serious level of sleep deprivation and poor sleep quality [6]. With these concerns, this study attempts to estimate the causal effect of sleep duration on mental health—specifically, anxiety levels—among young adults.

Findings of this study are expected to contribute to the development of effective sleep management strategies that promote mental health in the younger population. The remainder of this article is structured as follows. Section 2 presents the literature review and research question. Section 3 introduces the data and methodology, and Section 4 conducts the analysis. Drawing on the findings, Sections 5 and 6 respectively discuss the findings and conclude the paper.

## 2. Literature review and research questions

### 2.1. Lack of sleep and mental health

Sufficient sleep has been increasingly recognized as a fundamental determinant of mental health, shaping individuals’ ability to manage stress, emotions, and cognitive performance [7]. Furthermore, inadequate or irregular sleep patterns are not merely short-term inconveniences but risk factors that can be accumulated over time, leading to psychological distress and, in many cases, long-lasting mental health disorders. This has been further emphasized in the literature on youth and young adults, a population that is particularly susceptible to developmental challenges.

In particular, this phenomenon was examined among young adults, highlighting how insufficient sleep can increase stress sensitivity and emotional dysregulation [1]. Similarly, it was argued that sleep deprivation during adolescence is strongly associated with a wide range of adverse outcomes, including depression, anxiety, diminished self-esteem, reduced academic performance, and even suicidal ideation [2]. Such findings suggest that sleep is not simply a physiological necessity but also a protective factor for psychological resilience. Furthermore, psychological difficulties emerging during adolescence might persist into adulthood, manifesting in violent behaviors, antisocial tendencies, and repeated suicide attempts, thereby causing the long-term consequences of neglecting mental health maintenance during youth [3].

These previous studies converge on the notion that insufficient sleep in young people should be regarded as a critical public health concern rather than an individual lifestyle choice. Intervening at an early stage—through educational programs, institutional support, and policy initiatives—could reduce the incidence of sleep-related psychological vulnerabilities. Importantly, fostering sufficient and regular sleep patterns could serve as an important strategy for improving immediate well-being and safeguarding against chronic mental health disorders. Therefore, a systematic approach to inadequate sleep among young adults is essential for fostering healthier developmental trajectories and ensuring long-term psychological stability.

### 2.2. Drivers of sleep deprivation

Previous studies on the relationship between sleep duration and mental health have focused on sociodemographic and lifestyle factors such as age, gender, income, alcohol consumption, and smoking behavior. Much of this literature emphasizes correlational evidence, suggesting that sleep habits are closely intertwined with overall mental well-being, yet it often fails to disentangle underlying mechanisms. For example, a large-scale investigation was conducted using data from the Behavioral Risk Factor Surveillance System (BRFSS) [8]. They examined the association between insufficient sleep and frequent mental distress. Their analysis incorporated a broad set of eight confounding variables, such as age, gender, race/ethnicity, education, income, marital status, smoking status, alcohol use, and loss of health insurance. Such results provided that inadequate sleep was related to higher levels of psychological distress across diverse demographic groups [9].

However, those studies heavily relied on self-reported survey data, which might be subject to recall bias and subjective interpretation. Moreover, the lack of objective measures, such as biological or clinical indicators, limited the ability to establish more precise causal inferences. Laboratory-based studies are necessary to identify biological pathways that explain how sleep deprivation affects health outcomes. For example, changes in plasma metabolites under strictly controlled sleep deprivation conditions have been investigated, and several physiological markers associated with insufficient sleep were identified [10].Their work provides important evidence that biological processes are disrupted when sleep is curtailed. As a result, their study raised the question of how such physiological aspects translate into psychological consequences.

Recent studies have attempted to bridge these gaps by integrating both subjective and objective dimensions of sleep research. For instance, it was emphasized that sleep is a critical biobehavioral process influencing immune regulation and inflammation, both of which are closely related to mental health disorders [11]. Partial sleep deprivation in adolescents impaired cognitive and emotional performance, and it indicated that the interplay of behavioral and physiological mechanisms was crucial [12,13]. Such findings necessitate the importance of combining survey-based sociodemographic data with biological indicators such as hormonal measures, inflammatory markers, or neuroimaging evidence. Those approaches could advance understanding beyond simple correlations and toward establishing causal pathways between insufficient sleep and mental health deterioration.

### 2.3. Research issue

Previous research has been somewhat insufficient for analyzing the relationship between sleep deprivation and mental health in terms of physiological and nutritional aspects [14]. This study incorporates physiological and nutritional indicators as confounding variables to estimate the causal effect of sleep duration on mental health. Specifically, mental health as outcomes are further categorized into “normal” and “abnormal” based on established clinical criteria. Moreover, these interactions could be used to examine the association between sleep duration and mental health. It is expected that findings of this study could have significant implications for both academia and daily life, enhancing mental health of youth.

## 3. Data and methods

This study employed meta learners to estimate causal effects, which could be differently estimated for a specific treatment. This approach has been widely adopted in fields such as mobile advertising, social psychology, and business analytics [15]. Among various models from Meta-Learners, we adopted the S-Learner and T-Learner. While the S-Learner utilizes a single model to learn treatment and outcome jointly, it often underestimates treatment effects. On the other hand, T-Learner has demonstrated robust performance in prior research and is recognized for its efficiency in estimating heterogeneous treatment effects.

Then, we used raw data from the Korea National Health and Nutrition Examination Survey for 2022–2023, provided by the Korea Disease Control and Prevention Agency [16]. The dataset has variables from three components: the Health Interview Survey (298 variables), the Health Examination Survey (248 variables), and the Nutrition Survey (83 variables). For this study, the data were filtered to include young adults aged 19–34, with 1,405 participants after removing rows with missing values.

Specifically, sleep duration was defined as the treatment variable, and mental health status, measured by GAD-7, was the outcome (Y). Physiological indicators (diabetes, cholesterol, triglycerides, AST, creatinine category, and anemia) were treated as confounders because they could influence both sleep duration and mental health.

**Fig 1:**
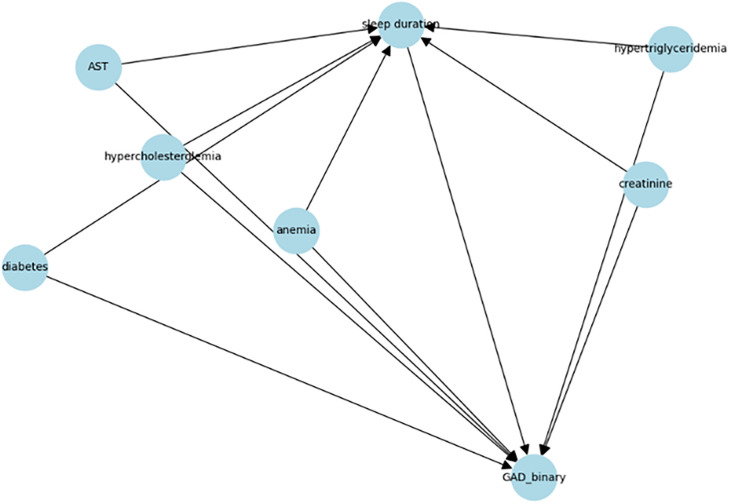
Causal DAG for physiological.

As shown in Fig 1, a causal directed acyclic graph (DAG) illustrates the identifying assumptions for estimating the effect of sleep duration on mental health status. Physiological indicators are treated as pre-treatment confounders that may influence both sleep duration and mental health.

**Fig 2:**
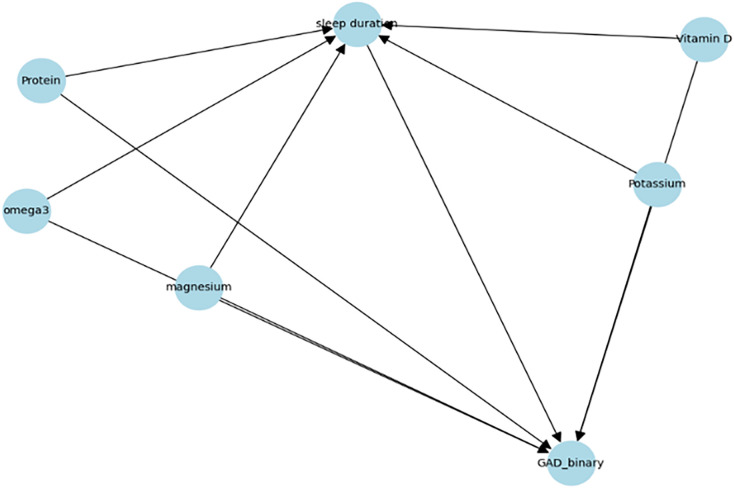
Causal DAG for nutritional.

In Fig 2, a causal directed acyclic graph (DAG) illustrates the identifying assumptions for estimating the effect of sleep duration on mental health status. Nutritional indicators are also treated as pre-treatment confounders that may influence both sleep duration and mental health.

Based on the assumed causal DAG, these variables were jointly adjusted to block backdoor paths between sleep duration and mental health status and to reduce confounding bias. All confounders were incorporated as covariates in the meta-learner framework to minimize confounding bias and satisfy the conditional ignorability assumption. No post-treatment variables were included in the adjustment set. Given the cross-sectional nature of the data, it was acknowledged that some variables could also lie along post-treatment pathways; therefore, the interpretation relies on the pre-treatment confounder assumption, which could be further discussed as a limitation.

The treatment variable was sleep duration, measured as the average weekday sleep time. Following the previous criteria, sleep duration was binarized into “insufficient” (≤6 hours) and “adequate” (7–8 hours) categories [17]. Cases of excessive sleep were excluded to focus the analysis on sleep deprivation. The outcome variable, mental health status, was assessed using the Generalized Anxiety Disorder-7 (GAD-7) scale. Then, based on guidelines from the National Center for Trauma, GAD-7 scores were dichotomized into “anxious” (≥5 points) and “normal” (≤4 points). Furthermore, we controlled for six physiological health indicators obtained: diabetes, hypercholesterolemia, hypertriglyceridemia, AST (SGOT), anemia, and blood creatinine level. These variables served as confounders, potentially influencing both sleep duration and mental health [18].

Table 1 presents descriptive statistics for the physiological variables. It shows relatively low average prevalence of hypercholesterolemia (mean = 0.07) and hypertriglyceridemia (mean = 0.08), indicating that these metabolic conditions were present in a small proportion. The diabetes indicator exhibits limited variability (mean = 1.14, SD = 0.39), reflecting categorical coding with most observations concentrated at the lower levels. Liver enzyme levels (AST) display substantial dispersion (mean = 19.90, SD = 11.90), with a wide observed range (8.00 to 186.00), suggesting heterogeneity in hepatic function across individuals. Creatinine levels show low mean values with moderate variability (mean = 0.07, SD = 0.25), while anemia status demonstrates relatively higher average values (mean = 0.78) with limited dispersion, indicating that anemia-related measurements were common and relatively stable within the sample.

**Table 1:** Descriptive Statistics for Physiological Variables.

	diabetes	hypercholesterolemia	hypertriglyceridemia	AST	creatinine	anemia
mean	1.14	0.07	0.08	19.90	0.07	0.78
std	0.39	0.26	0.27	11.90	0.25	0.16
min	1.00	0.00	0.00	8.00	0.00	0.36
max	3.00	1.00	1.00	186.00	1.00	1.38

Table 2 summarizes the descriptive statistics for the nutritional variables. Average intake levels varied considerably across nutrients, with magnesium showing a mean value of 76.08 and substantial variability (SD = 42.19). Omega-3 intake exhibited a relatively low mean (1.76) with moderate dispersion (SD = 1.41), whereas vitamin D intake demonstrated high variability across individuals (mean = 2302.12, SD = 1075.98), spanning a wide range from 267.26 to 8410.44. Protein intake also showed considerable heterogeneity (mean = 254.02, SD = 123.09), indicating marked differences in dietary protein consumption within the sample. Potassium intake displayed the most significant relative dispersion (mean = 2.72, SD = 5.12), with values ranging from 0.00 to 87.61, suggesting substantial inter-individual variation in potassium-related nutritional status.

**Table 2:** Descriptive Statistics for Nutritional Variables.

	magnesium	omega3	Vitamin D	Protein	Potassium
mean	76.08	1.76	2302.12	254.02	2.72
std	42.19	1.41	1075.98	123.09	5.12
min	8.05	0.06	267.26	27.35	0.00
max	395.12	12.82	8410.44	1117.99	87.61

**Fig 3:**
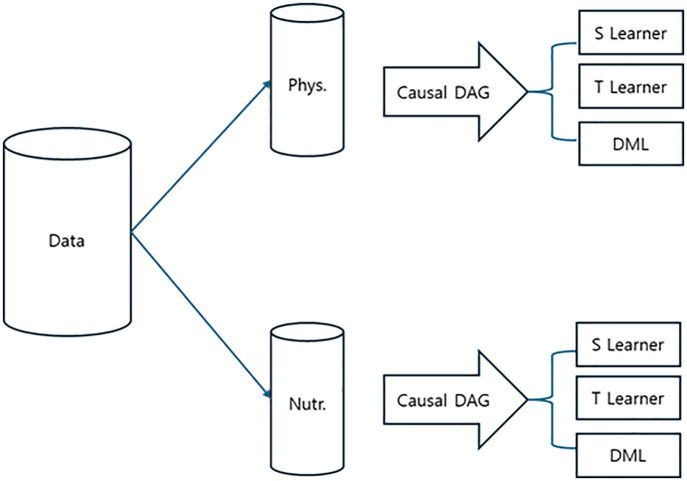
Analysis Flow.

Fig 3 illustrates the overall analytical workflow employed in this study. The raw dataset was first partitioned into two covariate sets representing physiological and nutritional factors, respectively.

For each covariate domain, a causal directed acyclic graph (DAG) was constructed to explicitly define the assumed causal structure. Based on these DAGs, three complementary causal estimation approaches—S-learner, T-learner, and Double Machine Learning (DML)—were subsequently applied in parallel to estimate the effect of sleep duration on anxiety outcomes. This stepwise framework allows for systematic comparison across different confounder specifications and estimation strategies, providing a coherent structure for evaluating robustness and consistency of the results.

The physiological and nutritional indicators as confounding variables were initially continuous measures, and those were categorized according to established clinical criteria and dietary intake levels. This could distinguish heterogeneous responses associated with specific biological states, to enhance the interpretability of the estimated causal relationship between sleep duration and mental health outcomes. For example, about hepatic function, aspartate aminotransferase (AST) levels are generally considered normal within the range of 0–40 IU/L, with values exceeding 40 IU/L indicating a high probability of liver damage [19]. Accordingly, AST was dichotomized into “normal (0)” for ≤40 IU/L and “abnormal (1)” for >40 IU/L. This is consistent with national health screening reference values and is clinically valid as an early screening threshold for liver dysfunction in young adults. Serum creatinine, an indicator of renal function, is typically regarded as normal within the range of 0.5–1.4 mg/dL [20]. In this study, values within this range were categorized as “normal (1),” while <0.5 mg/dL was classified as “low (0)” and >1.4 mg/dL as “high (2).” This three-level categorization reflects conditions such as reduced muscle mass or nutritional deficiency (“low”) and possible renal impairment (“high”), thus enabling a more nuanced analysis than a simple dichotomy.

In addition, for nutritional confounders, categorization was based on the Korea National Health and Nutrition Examination Survey and the Korean Nutrition Society. Protein intake was classified as “low (0)” for <48 g, “adequate (1)” for 48–72 g, and “high (2)” for >72 g, reflecting the recommended daily intake of 0.8–1.2 g/kg body weight for a 60-kg adult, necessary for muscle maintenance and neurotransmitter metabolism [21,22]. For omega-3 fatty acids (n-3), an upper threshold of 1 g was applied based on prior research [23,24]. Intake <0.2 g was categorized as “low (0),” 0.2–1 g as “adequate (1),” and >1 g as “high (2).” These thresholds are consistent with meta-analyses of Asian populations. Potassium (K) and magnesium (Mg) intakes were standardized to recommended target levels of 3,500 mg and 266.7 mg, respectively [25]. Using individual-level deviations from these targets, intake was categorized as “low (0)” if more than one standard deviation below, “high (2)” if more than one standard deviation above, and “adequate (1)” otherwise. This standard-deviation-based approach accommodates inter-individual differences in diet, body weight, and activity levels for improving empirical flexibility. Finally, Vitamin D status was determined using serum 25(OH)D levels, categorized as “deficient (0)” for <10 ng/mL, “adequate (1)” for 10–100 ng/mL, and “excess (2)” for >100 ng/mL [26,27]. Such cut-offs indicate widely used thresholds for deficiency and toxicity.

Then, the causal effects of sleep duration on mental health were estimated by meta-learner methods on a treatment variable (T), an outcome variable (Y), and a set of covariates (X) that might confound the relationship between T and Y. The S-Learner jointly models covariates (X) and the treatment variable (T) within a single predictive function to estimate outcomes by using discrete treatment variable. In addition, the T-Learner is further trained to estimate the Individual Treatment Effect (ITE). Specifically, the T-Learner was implemented by partitioning the data into treatment (T = 1) and control (T = 0) groups, and separately training conditional outcome models for each subset. Those models were compared to each other’s performance to estimate the causal effects. Each learner was combined with Random Forest and XGBoost to construct the predictive model. Random Forest and XGBoost are widely used ensemble algorithms. The causal effect was, then, estimated as the difference between the predicted outcomes of the two models. As the T-Learner is typically applied to binary treatment variables, sleep duration is transformed into a discrete variable by designating a reference category of 5 hours of sleep (control group). Each model was trained to estimate the conditional differences in mental health outcomes associated with variations in sleep duration.

As a result, four causal models were estimated by applying XGBoost and Random Forest to both the S-Learner and T-Learner approaches. Each model was used to estimate the causal effect of sleep duration on mental health, and the best-performing model was chosen. The performance of the causal inference models was evaluated using the area under the uplift curve (AUUC), which is not typical of the previous classification algorithm. AUUC could directly assess the discriminative ability of models to estimate individual treatment effects. The AUUC is calculated by ranking observations by their predicted uplift scores, plotting the cumulative observed treatment effects along the uplift curve, and computing the area under this curve. A higher AUUC indicates that the model more effectively identifies subgroups with substantial causal effects and demonstrates its capacity to capture heterogeneous treatment effects.

Specifically, uplift effects were estimated using sleep duration as a discrete treatment variable. A sleep duration of 5 hours was defined as the reference, and higher sleep duration levels were considered treatment groups for constructing the uplift curve. This could explain how accurately the models predicted the individualized effects of changes in sleep duration on mental health outcomes using AUUC. Accordingly, the AUUC scores were compared across four causal estimation models: S-Learner and T-Learner combined with XGBoost or Random Forest. Then, the model with the highest AUUC score was selected for understanding causal effects. Finally, to enhance causal inference, Double Machine Learning (DML) is applied to flexibly model both the outcome and treatment assignment mechanisms while orthogonalizing treatment effect estimation.

## 4. Results

### 4.1. Model with physiological confounders

First, findings on physiological factors were presented in Table 3. The model, combined with the T-Learner and Random Forest, achieved the highest performance, with an AUUC of 0.7833. This indicated that the model effectively identified individuals with substantial treatment effects, capturing variations in mental health associated with sleep duration. These findings also demonstrated the model's superior causal discrimination capability compared to others. As the T-Learner trains separate predictive models for each treatment group, findings could compare the treatment variable by examining the average ITE among different sleep durations.

**Table 3:** Comparison of AUUC Scores.

S-Learner		T-Learner	
Model	Score	Model	Score
XGBoost	0.6576	XGBoost	0.6802
Random-Forest	0.7742	Random-Forest	0.7833

As shown in Table 4, with 5 hours of sleep set as the control group, we investigated how incremental increases in sleep duration (from 6 to 10 hours) influence the likelihood of mental health improvement. An increase in sleep from 5 to 6 hours resulted in an average ITE of approximately +0.0251, suggesting that extending sleep to 6 hours was associated with an average 2.5 percentage point increase in the probability of mental health improvement. The effect became more pronounced at 7 hours (+0.0809) and peaked at 8 hours (+0.1424), indicating substantial gains in mental health outcomes. However, further increases to 9 hours (+0.1246) and 10 hours (+0.0895) were associated with a decline in average ITE, suggesting a potential tapering of the beneficial effects at longer sleep durations.

**Table 4:** Comparison of Average ITE by Sleep Duration (Reference Group: 5 Hours).

Change in Sleep duration	ITE Change
5h → 6h	+0.0251
5h → 7h	+0.0809
5h → 8h	+0.1424
5h → 9h	+0.1246
5h → 10h	+0.0895

**Fig 4:**
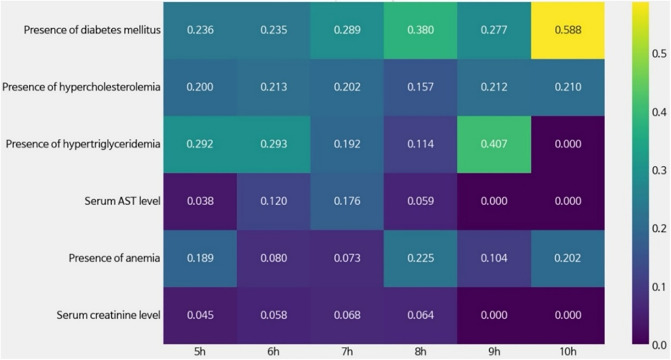
Variable Importance by T-Learner Model.

Fig 4 presents the relative feature importance of physiological covariates in predicting mental health outcomes, as estimated by T-Learner models trained separately for sleep durations ranging from 5 to 10 hours. This figure highlights that key physiological factors influencing mental health may vary with sleep duration.

Interestingly, the presence of diabetes mellitus consistently had high importance across all sleep duration models and recorded the highest feature importance (0.588) in the 10-hour sleep duration model. This could imply that the impact of diabetes status on mental health became more pronounced with longer sleep durations. Similarly, hypertriglyceridemia had high importance in the 5-hour and 6-hour models, declined at 8 hours, and then rose again at 9 hours (feature importance = 0.407). Interestingly, in the 10-hour model, the importance of hypertriglyceridemia dropped to zero, indicating a possible attenuation of its predictive contribution at extended sleep durations.

On the other hand, the serum AST level, serum creatinine level, and presence of anemia exhibited relatively lower feature importance compared to the other three variables. The presence of anemia contributed moderately in the 8-hour sleep model (feature importance = 0.225), suggesting that its influence might increase within specific sleep duration ranges. Furthermore, the importance of the other two features declined to 0 in the 9-hour and 10-hour models, suggesting that their impact on mental health outcomes may diminish as sleep duration increases.

### 4.2. Models with nutritional confounders

From a nutritional perspective, the predictive model of T-Learner with XGBoost achieved the highest performance, yielding an AUUC score of 0.8448, as shown in Table 5.

**Table 5:** Comparison of AUUC Scores.

S-Learner		T-Learner	
Model	Score	Model	Score
XGBoost	0.2848	XGBoost	0.3057
Random-Forest	0.8448	Random-Forest	0.7380

This table also indicates that the model effectively identified individuals with substantial treatment effects, capturing variations in mental health outcomes associated with sleep duration. Random-forest based T-Learner could estimate the individualized causal effects of sleep duration on mental health.

As shown in Table 6, with 5 hours of sleep set as the control group, we assessed how incremental increases in sleep duration (from 6 to 10 hours) influenced the likelihood of mental health improvement. An increase in sleep from 5 to 6 hours resulted in an average ITE of approximately +0.0196, indicating that extending sleep to 6 hours was associated with an average 1.9 percentage increase in the probability of improved mental health. The beneficial effect continued to rise at 7 hours (+0.0491) and 8 hours (+0.1259), demonstrating a gradual increase in mental health outcomes. Though a slight decrease was observed at 9 hours (+0.1089), the average ITE remained relatively high. At 10 hours (+0.1376), the effect increased again, indicating the improvement in mental health within this range. These findings suggest that sleep exerts a positive influence on mental health up to a certain threshold, with effects peaking at 8 and 10 hours of sleep.

**Table 6:** Comparison of Average ITE by Sleep Duration (Reference Group: 5 Hours).

Change in Sleep duration	ITE Change
5h → 6h	+0.0196
5h → 7h	+0.0491
5h → 8h	+0.1259
5h → 9h	+0.1089
5h → 10h	+0.1376

**Fig 5:**
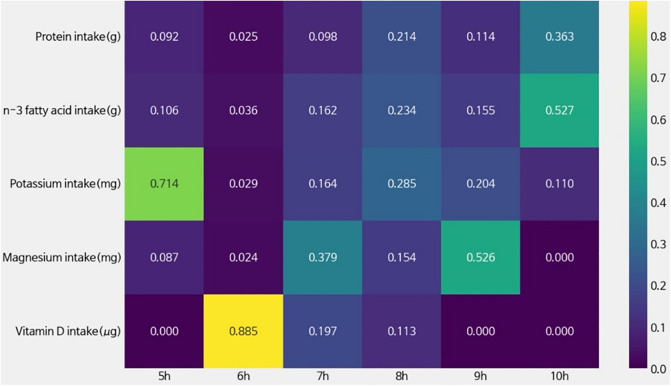
Variable Importance by T-Learner Model.

Fig 5 illustrates the feature importance of key nutritional factors in predicting mental health outcomes, as estimated by T-Learner models trained separately for each sleep duration range. The DML estimates showed a statistically significant average treatment effect of sleep duration on mental health after adjusting for physiological confounders (ATE = 0.034, 95% CI: 0.013–0.055) and nutritional confounders (ATE = 0.031, 95% CI: 0.011–0.052), demonstrating robustness of the findings to alternative confounder specifications.

Overall, Table 7 compares estimated treatment effects of increased sleep duration across physiological- and nutritional-adjusted models using T-learner and DML approaches. The T-learner results report individualized treatment effects (ITE) for incremental increases in sleep duration from a 5-hour baseline, showing progressively larger estimated effects up to 8 hours in both adjustment settings, followed by attenuated or variable effects at longer durations. The average treatment effects (ATEs) derived from the T-learner were similar across confounder sets (physiological ATE = 0.0925; nutritional ATE = 0.0891). In contrast, the DML approach yielded smaller but consistent ATE estimates per additional hour of sleep, with values of 0.0345 under physiological adjustment and 0.0314 under nutritional adjustment. The table highlights both the dose-specific patterns captured by the T-learner and the more conservative, average marginal effects estimated by DML, enabling direct comparison across methods and confounder specifications.

**Table 7:** Comparison of Treatment Effect.

Physiological				Nutritional			
T-Learner	ITE	5h → 6h	0.0251	T-Learner	ITE	5h → 6h	0.0209
		5h → 7h	0.0809			5h → 7h	0.05
		5h → 8h	0.1424			5h → 8h	0.1269
		5h → 9h	0.1246			5h → 9h	0.1101
		5h → 10h	0.0895			5h → 10h	0.1377
	ATE		0.0925		ATE		0.0891
DML	ATE		0.03447	DML	ATE		0.0314

The results indicated that the nutrients contributing to mental health could vary with sleep duration. First, Vitamin D exhibited the highest feature importance (0.885) in the 6-hour sleep model, but its importance declined or approached zero in other sleep duration models. This suggested that Vitamin D intake might be relevant to mental health under conditions of shorter sleep duration. Second, potassium demonstrated the most significant importance (0.714) in the 5-hour sleep model, with a gradual decrease in importance as sleep duration increased. Such a finding implied that potassium intake could play a critical role in predicting mental health outcomes during sleep deprivation. Third, n-3 fatty acid demonstrated a relatively consistent importance across sleep duration models, with its highest feature importance observed in the 10-hour sleep model (0.527). It suggested that n-3 fatty acid could play a significant role in predicting mental health outcomes under conditions of prolonged sleep. Fourth, magnesium was important in the 7-hour (0.379) and 9-hour (0.526) models but dropped to 0 in the 10-hour model. Also, protein showed a gradual increase in importance as sleep duration lengthened, reaching a contribution of 0.3627 in the 10-hour model. In summary, the key nutritional factors influencing mental health might differ depending on sleep duration.

## 5. Discussion

This study attempted to estimate how the physiological or nutritional factors influence the causal effects of sleep duration on mental health. Some causal inference models were constructed and compared using physiological and nutritional variables. This comparison was performed using AUUC metrics, ITE changes, feature importance, and DML. As a result, the T-Learner, combined with Random Forest and incorporating physiological variables as covariates, achieved an AUUC of 0.7380. Furthermore, the model based on nutritional variables (T-Learner with Random Forest) recorded an AUUC of 0.7833. It indicated that the nutrition–based model was more effective at distinguishing individuals with substantial treatment effects.

ITE changes revealed both similarities and differences between the two models. In both cases, the estimated causal effects of sleep duration on mental health increased with longer sleep duration, then plateaued or declined, reflecting a nonlinear relationship. Specifically, the physiological model exhibited the highest average ITE (+0.1424) at 8 hours of sleep, after which the effect decreased. In contrast, the nutritional model continued to increase, reaching its maximum ITE (+0.1376) at 10 hours. Furthermore, the incremental ITE from 5 to 6 hours of sleep was slightly larger in the physiological model (+0.0251) than in the nutritional model (+0.0196), suggesting that the physiological model captured ITE changes with greater sensitivity. The physiological model consistently identified diabetes as an important feature across all sleep duration ranges, with the highest importance (0.589) in the 10-hour sleep duration model. In contrast, the nutritional model identified Vitamin D intake as the most prominent feature (importance = 0.885) in the 6-hour sleep model. Moreover, potassium intake was important at short sleep durations, while n-3 fatty acid intake was influential at long sleep durations.

Finally, DML provided that each additional hour of sleep was associated with a significant improvement in mental health (ATE = 0.034, 95% CI: 0.013–0.055). A similar effect size was observed when adjusting for nutritional factors (ATE = 0.031, 95% CI: 0.011–0.052), indicating the robustness of the estimated causal effect across different confounder sets. In addition, T-learner analyses revealed a nonlinear dose–response pattern, with the most considerable marginal benefits observed between 5 and 8 hours of sleep, followed by diminishing returns at longer durations. Uplift evaluation metrics (QINI and AUUC) further suggested meaningful heterogeneity in treatment effects, particularly in the nutritional model.

Findings from this study suggest a significant causal association between insufficient sleep and increased GAD-7 scores, indicating a higher likelihood of elevated anxiety levels among young adults with short sleep duration. Particularly, the physiological model demonstrated a clearer association between sleep duration and improvements in mental health. In contrast, the nutritional model showed superior discriminative performance as measured by AUUC. The feature importance revealed that the choice of variables could substantially influence causal inference outcomes for the same treatment variable (sleep duration). This result supports that sleep deprivation could deteriorate mental health of youth. Furthermore, the observed associations between insufficient sleep and physiological factors, particularly the prevalence of hypercholesterolemia and elevated AST (SGOT) levels, suggest that sleep may also influence broader aspects of physical health.

Finally, findings could contribute to current efforts to raise public awareness and policy focus on sleep hygiene among youth. The discovered relationship between sleep duration and mental health outcomes necessitates the importance of promoting healthier sleep patterns as a strategy to enhance psychological resilience in this demographic. However, it is necessary to interpret these results carefully. While causal inference methods were applied, the potential for reverse causality and the influence of unmeasured confounders not accounted for in this study remain. These limitations indicate the need for further efforts, employing longitudinal or experimental designs, to validate the direction and robustness of the observed effects.

Furthermore, although the present study provides empirical causal evidence that sleep duration is associated with improvements in mental health outcomes after adjusting for physiological and nutritional factors, it does not explicitly test a theory-driven mechanism linking sleep deprivation to metabolic abnormalities or nutritional deficiencies. Future research could integrate established biological and behavioral theories into a unified causal framework. In particular, longitudinal or panel data would allow researchers to distinguish whether metabolic and nutritional factors act primarily as predisposing conditions, mediators, or feedback mechanisms in the sleep–mental health relationship. Such extensions would contribute to a deeper theoretical understanding of how sleep deprivation translates into metabolic and nutritional imbalance and adverse mental health outcomes.

In particular, the present study adopts a meta-learner and DML-based causal inference that jointly accounts for physiological and nutritional confounders, thereby distinguishing it from previous work. Our results are broadly consistent with prior epidemiological studies reporting that shorter sleep duration is associated with poorer mental health outcomes. Although differences in study design, populations, and analytical methods preclude direct quantitative comparison of previous studies, the alignment of effect directions provides indirect support for the validity of our findings. Future studies could build on this work by conducting head-to-head comparisons of causal inference approaches with traditional statistical models, as well as by replicating the analysis across different age groups or longitudinal settings.

## 6. Conclusion

This study was motivated by the need to examine the causal relationship between sleep duration and mental health, with particular attention to how different types of covariates—physiological and nutritional indicators—influence causal inference outcomes. We proposed an approach for handling and analyzing individualized treatment effects that integrates both physiological and nutritional variables with use of meta learners and uplift modeling. This effort could bring a more nuanced estimation of treatment effect heterogeneity, providing insights not only into the overall association between sleep and mental health but also into the role of covariate factors in estimating causal effects. In addition, it is necessary to consider the characteristics and temporal dynamics of covariates to improve model reliability and deepen understanding of how sleep duration interacts with physiological and nutritional factors to affect mental health. Future studies could adopt a more comprehensive approach to achieve more accurate and personalized implications regarding sleep and mental health.
